# Response Efficacy of PD-1 and PD-L1 Inhibitors in Clinical Trials: A Systematic Review and Meta-Analysis

**DOI:** 10.3389/fonc.2021.562315

**Published:** 2021-04-16

**Authors:** Shixue Chen, Zhibo Zhang, Xuan Zheng, Haitao Tao, Sujie Zhang, Junxun Ma, Zhefeng Liu, Jinliang Wang, Yuanyu Qian, Pengfei Cui, Di Huang, Ziwei Huang, Zhaozhen Wu, Yi Hu

**Affiliations:** ^1^ Department of Medical Oncology, Chinese People’s Liberation Army (PLA) General Hospital, Beijing, China; ^2^ Department of Graduate Administration, Chinese PLA General Hospital, Beijing, China; ^3^ Department of Cardiothoracic Surgery, The 78^th^ Group Army Hospital of Chinese PLA, Mudanjiang, China; ^4^ School of Medicine, Nankai University, Tianjin, China

**Keywords:** PD-1/PD-L1 inhibitors, meta-analysis, response efficacy, objective response rate, time to response, duration of response

## Abstract

**Background:**

Immune checkpoint inhibitors targeting the PD-1/PD-L1 pathway have demonstrated promise in treating a variety of advanced cancers; however, little is known regarding their efficacy under various clinical situations, including different cancer types, treatment lines, drug combinations, and therapeutic regimens.

**Methods:**

Published articles and conference abstracts (in English) in PubMed, Embase, the Cochrane Central Register, and Web of Science were searched up to February 10, 2020. The data were analyzed by the meta-analysis program in Stata.

**Results:**

A total of 16,400 patients from 91 clinical trials were included in this meta-analysis. PD-1/PD-L1 inhibitors had a mean ORR of 19.56% (95% CI: 15.09–24.03), a median TTR of 2.05 months (m) (95%CI: 1.85–2.26), and a median DOR of 10.65 m (95%CI: 7.78–13.52). First-line treatment had a higher ORR (36.57% vs. 13.18%) but a shorter DOR (9.00 m vs. 13.42 m) compared to the second-line or subsequent treatment. Immunotherapy combined with chemotherapy (I+C) (46.81% [95%CI: 36.02–57.60]) had a statistically significant higher ORR compared to immunotherapy (I) (17.75% [95%CI: 14.47–21.03]) or immunotherapy combined with immunotherapy (I+O) (12.25% [95%CI: 1.56–22.94]), while I+C (8.09 m [95%CI: 6.86–9.32]) appeared to reduce the DOR compared to I (12.39 m [95%CI: 7.60–17.18]). PD-1 inhibitors were associated with better ORR (21.65% vs. 17.60%) and DOR (11.26 m vs. 10.03 m) compared to PD-L1 inhibitors. There were no significant differences in TTR under different situations.

**Conclusions:**

PD-1/PD-L1 inhibitors were promising immunotherapeutic agents to achieve satisfactory response efficacies with different cancer types, treatment lines, drug combinations, and therapeutic regimens. This comprehensive summary of the response efficacy of PD-1/PD-L1 inhibitors serves as a reference for clinicians to make evidence-based decisions.

## Introduction

Cancer continues to be one of the most threatening diseases to human health and a leading cause of mortality worldwide (1). Some cancers are refractory to chemotherapy or targeted therapy. Blocking the immune checkpoint of programmed cell death-1 (PD-1) or programmed cell death-ligand 1 (PD-L1) receptor have led to great improvements in disease outcomes, and PD-1/PD-L1 inhibitors have emerged as frontline treatments for various cancers such as non-small cell lung cancer, metastatic melanoma, and renal cell carcinoma. PD-1 is a negative regulator with increased expression on reactive anti-tumor T cells, and its ligand PD-L1 is mainly expressed on the surface of tumor cells; the binding of PD-1 and PD-L1 can turn off the anti-tumor effect of T cells. Thus, PD-1/PD-L1 inhibitors can relieve immune suppression of anti-tumor T cells, which results in T cell proliferation, infiltration into the tumor microenvironment, and the induction of an anti-tumor response (2, 3). Unlike traditional anti-tumor treatments, PD-1/PD-L1 inhibitors have been reported to have a long-lasting anti-tumor response with reactivation of the immune system. While PD-1 and PD-L1 inhibitors have shown promise in advanced cancer immunotherapy, only a subset of patients can respond to this therapy, and the majority do not benefit. Thus, it is critical to understand the response efficacy of these drugs under various clinical situations.

Currently, two PD-1 inhibitors (nivolumab and pembrolizumab) and three PD-L1 inhibitors (atezolizumab, avelumab, and durvalumab) have been approved for the first-line or subsequent therapy of various cancer types. Hundreds of clinical trials of PD-1/PD-L1 inhibitors have reported response efficacy, including objective response rate (ORR: the proportion of patients experiencing complete response or partial response per RECIST v1.1 at any time during the study), time to response (TTR: the time from initiation of the treatment to the date of first documented complete or partial response), and duration of response (DOR: the time from first documented complete or partial response to disease progression or death). However, the reported response efficacies had substantial variations due to distinct clinical situations, such as different cancer types, drugs, treatment lines, and management practices among studies.

We therefore conducted a meta-analysis to comprehensively compare the response efficacy of PD-1/PD-L1 inhibitors in patients with a variety of cancer types, therapeutic regimens, treatment lines, and management strategies among published clinical trials.

## Materials and Methods

### Search Methods and Study Selection

We identified published clinical trials of PD-1/PD-L1 inhibitors that reported the response efficacy (ORR, TTR, and DOR) of advanced tumors from PubMed, Embase, the Cochrane Central Register, and Web of Science from their inceptions to February 10, 2020. The abstracts from major conference proceedings of the World Conference on Lung Cancer (WCLC), the American Society of Clinical Oncology (ASCO), the American Association for Cancer Research (AACR), and the European Society of Medical Oncology (ESMO) were also reviewed, with the search terms PD-1, PD-L1, PD-1 inhibitor, PD-L1 inhibitor, nivolumab, pembrolizumab, atezolizumab, avelumab, and durvalumab. We evaluated all search results according to the Preferred Reporting Items for Meta-Analyses (PRISMA) statement (4). Studies eligible for inclusion met all of the following criteria: (1) Clinical trials for advanced cancer therapy; (2) Patients were treated with a PD-1 or PD-L1 inhibitor; (3) Studies that reported at least one outcome of interest (ORR, TTR, or DOR); (4) Studies that were restricted to the English language. Letters, case reports, review articles, editorials, commentary articles, and expert opinions were excluded.

Data were extracted independently by three investigators (S.X. Chen, Z.B. Zhang, and X. Zheng) with a predefined information sheet. Any disagreements were discussed and resolved with a third investigator (P.F. Cui).

### Data Extraction

The data extracted from each included trial were the trial name or first author’s name, year of publication, name of publication, phase, cancer type, treatment line, stage, number of enrolled patients and responders, PD-1 and PD-L1 inhibitor used, drug management, ORR, TTR, DOR, and median follow-up time.

### Efficacy Evaluation

The study outcomes measured for response efficacy were ORR, TTR, and DOR. The treatment response in each clinical trial was determined by Response Evaluation Criteria in Solid Tumors (RECIST) version 1.1. ORR was recorded as a percentage. The time durations for TTR and DOR were recorded in unit of month.

### Main Outcomes

Response efficacy (ORR, TTR, and DOR) among different cancer types, treatment lines, drug combinations, and therapeutic regimens.

### Statistical Analysis

Meta-analyses were conducted to synthesize the pooled ORR, TTR, and DOR by different clinical conditions, including cancer types, treatment lines, drug combinations, and therapeutic regimens. The heterogeneity of different outcomes was assessed by Q and I^2^. Both inverse-variance fixed- and random-effects meta-analyses were explored for each comparator. When P > 0.1 and I^2^ < 50%, it indicated that the studies were homogeneous, and a fixed effect model was selected. Otherwise, a random effect model was used (P < 0.1 or I^2^ > 50%). A Dersimonian-Laird random effects model was used to account for both within- and between-study heterogeneity when substantial heterogeneity was observed (5). Quality assessment of included studies was performed using the Cochrane Risk of Bias Tool for RCTs. Publication bias was accessed by funnel plot and Begg’s and Egger’s tests.

The ORR proportions were transformed to a logit scale (logit (z) = log (z)-log (1-z)) to calculate the 95% confidence interval (CI), and then transformed back to proportions. For TTR and DOR, we used elementary inequalities and approximations to estimate the mean and standard deviation from the median, range and sample size by verified formulas, which were distribution-free of the underlying data (6–8).

For TTR, DOR, and ORR, we utilized forest plots to describe the values or incidences and their 95% CIs by cancer type, treatment line, drug combination, and therapeutic regimen. The data were analyzed in Stata (version 15.0) using the “metafor” package. Non-overlapping 95% CIs of different subgroups were considered statistically significant from the forest plots.

## Results

### Eligible Studies and Characteristics

**Figure 1:**
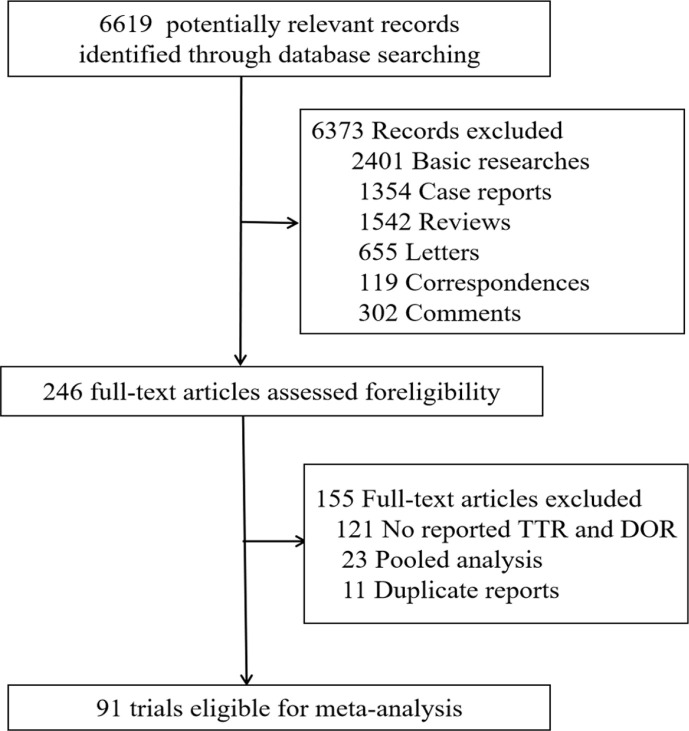
Flow diagram of the literature search and trial selection process.

A total of 6,619 relevant publications were identified from the database search, of which 6,373 were excluded for not meeting the inclusion criteria. Then, 246 publications were reviewed for full-text evaluation, of which 11 were duplicates and 121 reported neither available TTR nor DOR for analyses and were therefore excluded. In the end, a total of 91 clinical trials involving 16,400 patients were included in this meta-analysis (**Figure 1**) (9–98). The main characteristics of the 91 eligible trials are listed in **Table S1**. The numbers of trials for pembrolizumab, nivolumab, atezolizumab, avelumab, and durvalumab were 35, 33, 15, 4, and 4, respectively. The trials covered the treatments of various cancer types: 25 trials in lung cancer, 8 in melanoma, 19 in genitourinary cancer, 10 in head-and-neck cancer, 6 in breast cancer, 12 in gastrointestinal cancer, and 11 in other cancers (1 in skin cancer, 1 in brain cancer, 2 in Hodgkin lymphoma, 1 in soft-tissue sarcoma or bone sarcoma, 1 in thymic carcinoma, 2 in hepatocellular carcinoma, 1 in sarcoma, and 2 in malignant pleural mesothelioma). Among all selected trials, there were 19 trials for the first-line treatment, 51 for the second-line and subsequent treatments, and 23 trials covered the first-line as well as subsequent treatments. Eighty-three clinical trials were performed with single-agent PD-1/PD-L1 (I), 7 with PD-1/PD-L1 combined with chemotherapy (I+C), and 6 with PD-1/PD-L1 combined with other immune checkpoint inhibitors (I+O). In addition, 2 studies (17, 98) reported research data of first-line and non-first-line separately, and 5 studies (23, 26, 42, 54, 55) had both I and I+C therapy arms.

## ORR

### By Cancer Type

**Figure 2:**
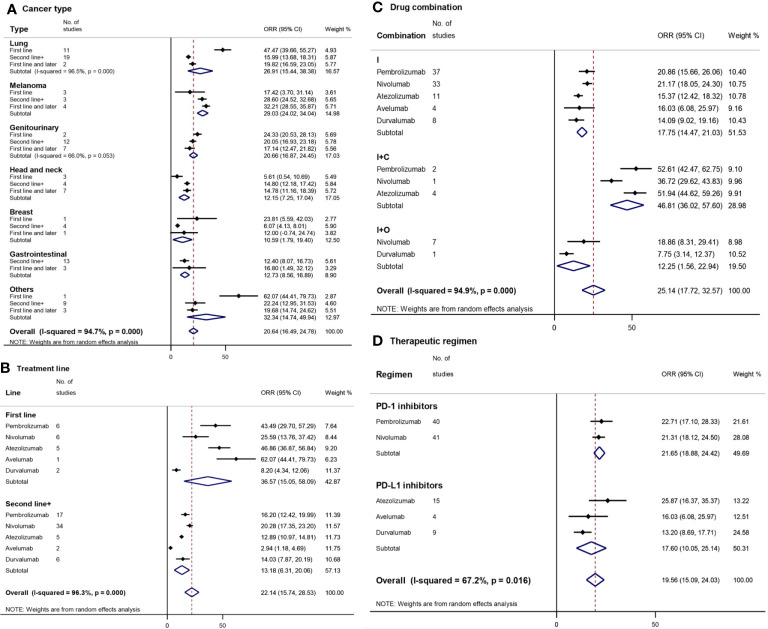
Forest plot for objective response rate (ORR) by cancer type **(A)**, treatment line **(B)**, drug combination **(C)**, and therapeutic regimen **(D)**. Squares represent the study-specific effect size (ORR). The area of the square is inversely proportional to the standard error of the study (and therefore indirectly to the sample size), and a larger area indicates greater weight in the calculation of the pooled effect size. The horizontal line crossing the square represents the 95% CI. The diamonds represent the estimated overall effect based on the meta-analysis. CI, confidence interval.

Based on the cancer type included in the 91 selected clinical trials, we divided these trials into 7 subgroups (**Figure 2A**). The subgroups with the highest ORR were observed in ‘others’ (32.34% [95%CI: 14.74–49.94]) and melanoma (29.03% [95%CI: 24.02–34.04]), followed by lung cancer (26.91% [95%CI: 15.44–38.38]) and genitourinary cancer (20.66% [95%CI: 16.87–24.45]). The mean ORR of melanoma was statistically significantly higher than that of the other three cancer types, gastrointestinal, head and neck, and breast cancer. Except for melanoma and head-and-neck cancer, the ORR of the first-line treatment was generally better than that of second-line or subsequent treatment, which had a statistically significant difference in ‘others’ and lung cancer. For the first-line treatment, the highest ORR was observed in ‘others’ (Merkel cell skin carcinoma, 62.07% [95%CI: 44.41–89.73]) and lung cancer (47.47% [95%CI: 39.66–55.27]), which was significantly higher than that of the other five cancer types.

### By Treatment Line

In order to compare the exact differences between the first-line treatment and the second-line or subsequent treatment, clinical trials (N = 23) simultaneously covering first-line as well as subsequent line treatment were excluded. The overall mean ORR of the first-line treatment (36.57% [95%CI: 15.05–58.09]) with PD-1/PD-L1 inhibitors was higher compared to the second-line or subsequent treatment (13.18% [95%CI: 6.31–20.06]) (**Figure 2B**). Pembrolizumab, atezolizumab, and avelumab had a statistically significantly higher ORR of the first-line treatment (43.49% [95%CI: 29.70–57.29], 46.86% [95%CI: 36.87–56.84], and 62.07% [95%CI: 44.41–79.73], respectively) compared to that of the second-line or subsequent treatment (16.20% [12.42–29.99], 12.89% [10.97–14.81], and 2.94% [1.18–4.69], respectively).

Avelumab and atezolizumab had the highest ORR among the first-line treatments, while nivolumab (20.28% [17.35–23.20]) and pembrolizumab had the highest ORR among the second-line or subsequent treatments.

### By Drug Combination

I+C (46.81% [95%CI: 36.02–57.60]) had a statistically significantly higher ORR compared to I (17.75% [95%CI: 14.47–21.03]) or I+O (12.25% [95%CI: 1.56–22.94]) (**Figure 2C**). Among I+C therapies, atezolizumab (51.94% [95%CI: 44.62–59.26]) had a statistically significantly higher ORR compared to nivolumab (36.72% [95%CI: 29.62–43.83]). No statistically significant differences were found in ORR between different drugs in each subgroup.

### By Therapeutic Regimen

The overall mean ORR of PD-1/PD-L1 inhibitors was 19.56% with a 95%CI of 15.09–24.03% (**Figure 2D**). The ORR of PD-1 inhibitors (21.65% [95%CI: 18.88–24.42]) was higher than that of PD-L1 inhibitors (17.60% [95%CI: 10.05–25.14]). Atezolizumab (25.67% [95%CI: 16.37–35.37]) and durvalumab (13.20% [95%CI: 8.69–17.71]) had the highest and lowest ORR, respectively.

## TTR

### By Cancer Type

**Figure 3:**
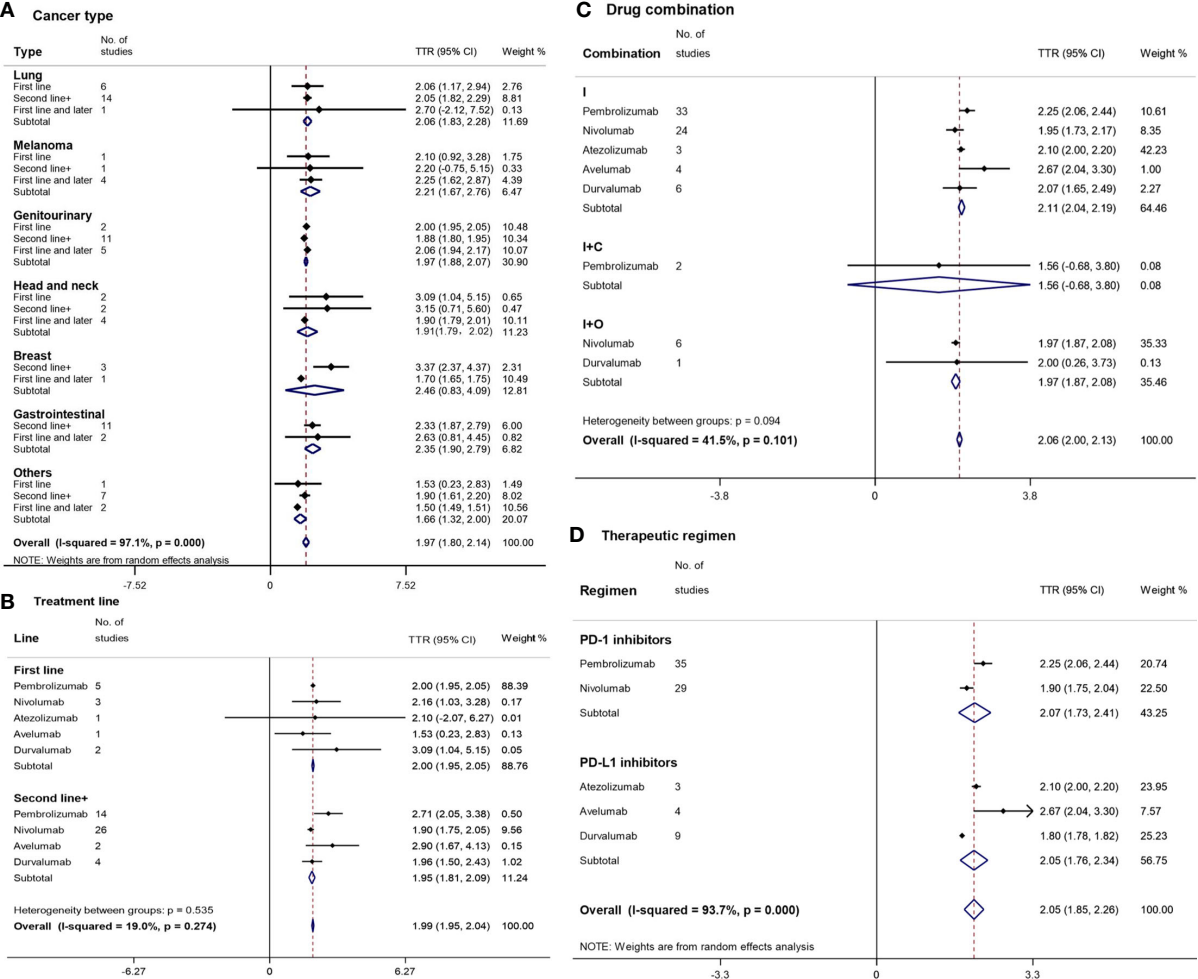
Forest plot for time to response (TTR) by cancer type **(A)**, treatment line **(B)**, drug combination **(C)**, and therapeutic regimen **(D)**. Squares represent the study-specific effect size (TTR). The area of a square is inversely proportional to the standard error of the study (and therefore indirectly to the sample size), and a larger area indicates greater weight in the calculation of the pooled effect size. The horizontal line crossing the square represents the 95% CI. The diamonds represent the estimated overall effect based on the meta-analysis. CI, confidence interval.

The shortest and longest TTR were found in ‘others’ (1.66 months (m) with a 95%CI of 1.32–2.00 m) and breast cancer (2.46 m [95%CI: 0.83–4.09]), respectively (**Figure 3A**), which were similar to the TTR of other cancer types. There were also no significant differences in TTR either between the first-line treatment and the second-line or subsequent treatments of each cancer type.

### By Treatment Line

The median TTR of PD-1/PD-L1 inhibitors in the first-line treatment was similar to that in the second-line treatment (**Figure 3B**). Except for nivolumab (1.90 m [95%CI: 1.75–2.05]), which had a shorter TTR than pembrolizumab (2.71 m [95%CI: 2.05–3.38]), there was no other statistically significant difference in TTR among various drugs and treatment lines. The shortest and longest TTR were found in avelumab (1.53 m [95%CI: 0.23–2.83]) and durvalumab (3.09 m [95%CI: 1.04–5.15]) of the first-line treatments, respectively.

### By Drug Combination

I+C (1.56 m [95%CI: 0.68–3.80]) had a shorter TTR than I+O (1.97 m [95%CI: 1.87–2.08]) and I (2.11 m [95%CI: 2.04–2.19]) without a statistically significant difference (**Figure 3C**). Nivolumab (1.95 m [95%CI: 1.73–2.17]) had the shortest TTR in the monotherapy subgroup.

### By Therapeutic Regimen

The overall median TTR of PD-1/PD-L1 inhibitors was 2.05 m with a 95%CI of 1.85 m to 2.26 m (**Figure 3D**). PD-1 inhibitors (2.07 m [95%CI: 1.73–2.41]) and PD-L1 inhibitors (2.05 m [95%CI: 1.85–2.26]) had a similar TTR. The TTRs of durvalumab (1.80 m [95%CI: 1.78–1.82]) and nivolumab (1.90 m [95%CI: 1.75–2.04]) were statistically significantly shorter than those of pembrolizumab (2.25 m [95%CI: 2.06–2.44]) and avelumab (2.67 m [95% CI: 2.04–3.30]).

## DOR

### By Cancer Type

**Figure 4:**
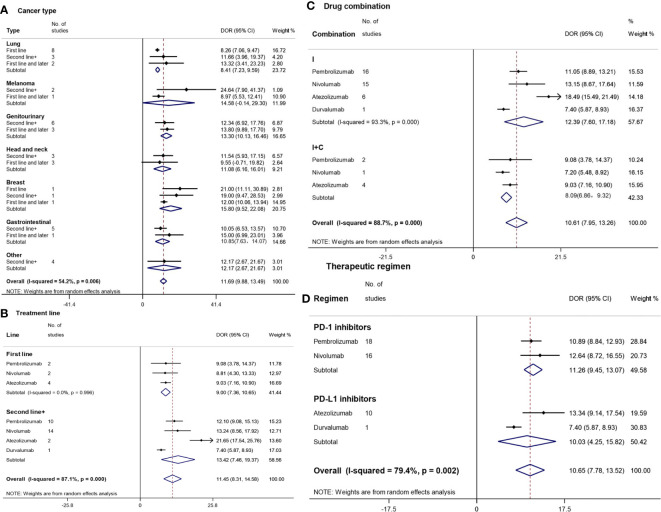
Forest plot for duration of response (DOR) by cancer type **(A)**, treatment line **(B)**, drug combination **(C)**, and therapeutic regimen **(D)**. Squares represent study-specific effect sizes (DOR). The area of a square is inversely proportional to the standard error of the study (and therefore indirectly to the sample size), and a larger area indicates greater weight in the calculation of the pooled effect size. The horizontal line crossing the square represents the 95% CI. The diamonds represent the estimated overall effect based on the meta-analysis. CI, confidence interval.

The longest DORs were observed for breast cancer (15.80 m [95%CI: 9.52–22.08]) and melanoma (14.58 m [95%CI: 0.14–29.30]), followed by genitourinary cancer (13.30 m [95%CI: 10.13–16.46]), ‘others’ (12.17 m [95%CI: 2.67–21.67]), head-and-neck cancer (11.08 m [95%CI: 6.16–16.01]), gastrointestinal cancer (10.85 m [95%CI: 7.63–14.07]), and lung cancer (8.41 m [95%CI: 7.23–9.59]) (**Figure 4A**). There were no statistically significant differences in DOR between various cancer types except for the difference between genitourinary cancer and lung cancer. In addition, no statistically significant differences were found between different treatment lines in each cancer type.

### By Treatment Line

The DOR of the second and subsequent treatments (13.42 m [95%CI: 7.46–21.67]) was unexpectedly longer than that of the first-line treatment (9.00 m [95%CI: 7.36–10.65]) (**Figure 4B**). The longest and shortest TTRs were found in atezolizumab (21.65 m [95%CI: 17.54–25.76]) and durvalumab (7.40 m [95%CI: 5.87–8.93]) among the second and subsequent treatments, respectively, which had a statistically significant difference.

### By Drug Combination

Patients treated with I (12.39 m [95%CI: 7.60–17.18]) had a longer DOR compared with those treated with I+O (8.09 m [95%CI: 6.86–9.32]), though there was no significant difference (**Figure 4C**). In the monotherapy subgroup, the longest DOR was observed for patients treated with atezolizumab (21.65 m [95%CI: 17.54–25.76]), followed by those treated with nivolumab (13.15 m [95%CI: 8.67–17.64]), pembrolizumab (11.05 m [95%CI: 8.89–13.21]), and durvalumab (7.40 m [95%CI: 5.87–8.93]).

### By Therapeutic Regimen

The overall median DOR of PD-1/PD-L1 inhibitors was 10.65 m (95%CI: 7.78–13.52) (**Figure 4D**). PD-1 inhibitors (11.26 m [95%CI: 9.45–13.07]) and PD-L1 inhibitors (10.03 m [95%CI: 4.25–15.82]) had similar TTRs. The longest DOR was observed for atezolizumab (13.34 m [95%CI: 9.14–17.54]), followed by nivolumab (12.64 m [95%CI: 8.72–16.55]), pembrolizumab (10.89 m [95%CI: 8.84–12.93]), and durvalumab (7.40 m [95%CI: 5.87–8.93]).

### Publication Bias

Quality assessment of the 28 included RCTs was performed using the Cochrane Risk of Bias Tool (**Figure S1**). Due to lack of appropriate evaluation tools, the risk of bias of the 63 single arm clinical trials was not estimated. Funnel plots (**Figures S2** **–** **4**), Begg’s tests, and Egger’s tests (**Table S2**) for the majority of treatments demonstrated symmetrical results (P > 0.05), while a few treatments were asymmetrical due to the heterogeneity of the drugs and doses, intention to treat populations, or lack of contrast cohorts in some studies. However, this meta-analysis is still suggested to be sufficiently effective considering the following: (1) it included a large number of high-quality studies; (2) we conducted subgroup analysis under various clinical situations; and (3) we adopted statistical methods with high test efficiencies. Further prospective, randomized control trials are warranted to reduce publication bias and for validation.

## Discussion

Targeting the PD-1 and PD-L1 pathway is an important new approach to cancer therapy. PD-1/PD-L1 inhibitors can reactivate T cells to work against cancer cells, though they depend on pre-existing anti-tumor T cells. The mechanism of response involves infiltrating T cells and engaging their receptors to recognize tumor antigens and trigger the expression of PD-1 on T cells and PD-L1 on cancer cells; this can be simply reversed by blocking PD-1/PD-L1 (99). Anti-PD-1/PD-L1 treatments are optional for cancer patients, and only a portion of patients benefit from this immunotherapy. Patients may lack immune activation factors such as tumor antigens and pre-existing anti-tumor T cells (CD8^+^T cells) in the tumor, which may weaken the anti-tumor effect of PD-1/PD-L1 inhibitors. PD-1 blockade is unlikely to work if there are no CD8^+^T cells to be inhibited by PD-1 and PD-L1 interaction in the tumor microenvironment (100, 101).

Immune-combination therapies are often used to improve anti-tumor efficacy. In addition to the combination of chemotherapy or targeted therapy with PD-1/PD-L1 inhibitors, promising therapeutic options, such as the combination of oncolytic viruses, have shown anti-tumor activity for patients who fail to respond or achieve durable responses following immunotherapy (102–104). Oncolytic viruses are tumor specific and have the advantage of triggering anti-tumor immune responses in the tumor microenvironment, so the combination of oncolytic viruses and PD-1/PD-L1 inhibitors may be a useful strategy for future cancer treatment (102–104).

Due to the reactivation of the immune system and the prolonged response once immunotherapy works, clinicians are interested in response efficacy metrics (ORR, TTR, and DOR) other than overall survival (OS, time from initiation of the treatment to death from any cause) and progression-free survival (PFS, time from initiation of the treatment to disease progression or death from any cause, whichever occurred first). The emerging use of PD-1/PD-L1 inhibitors in clinical practice highlights an urgent need for evidence-based analysis to understand their exact response efficacy. Data from multiple clinical trials suggest that the wide range of response efficacy can be attributed to either variability in therapeutic regimens or differences due to drug combinations. Other significant factors are the heterogeneity in distinct cancer types and treatment lines. This is the first meta-analysis to study the response efficacy of PD-1/PD-L1 inhibitors under various clinical situations, including cancer type, treatment line, therapeutic regimen, and medication combination. This comprehensive response efficacy analysis provides clinicians with an important reference and guidelines for making clinical decisions.

When pooling quantitative data in a meta-analysis, it is necessary to know the mean and standard deviation in every single study. However, obtaining original individual patient data from numerous clinical trials in order to calculate the means and standard deviations is difficult. Thus, this meta-analysis introduced a method of estimating the mean and standard deviation based on the median, range, and sample size (6, 7). Therefore, quantitative data without means and standard deviations can also be used in a meta-analysis without loss of relative accuracy.

Our pooled analysis showed that PD-1/PD-L1 inhibitors had a mean ORR of 19.56%, a median TTR of 2.05 m, and a median DOR of 10.65 m overall. The mean ORR ranged from 10.59% to 32.34% among various cancer types, while ‘others’, melanoma, and lung cancer had relatively high ORRs of about 30%. The highest ORR was 62% observed in ‘others’ from a study of Merkel cell skin carcinoma (27). The DORs for breast cancer and melanoma lasted about 15 months, approximately twice the DOR for lung cancer (8.41 m), but was not significantly different due to the overlapping 95% CI. These results indicate that PD-1/PD-L1 inhibitors can induce effective anti-tumor responses in a wide range of tumor types.

PD-1/PD-L1 inhibitors had a higher ORR for the first-line treatment compared to the second-line or subsequent treatment (36.57% vs. 13.18%). In the first-line treatment, avelumab (62.07%), atezolizumab (46.86%), and pembrolizumab (43.49%) showed favorable ORRs of over 40%. In addition, the inclusion of clinical studies using immunotherapy combined with chemotherapy as the first-line treatment was one reason for the improvement of ORR. Unlike ORR, the DOR of second-line or subsequent treatments was unexpectedly 4.42 m better than that of the first-line treatment (13.42 m vs. 9.00 m). This suggests that immunotherapy can sustain a prolonged response regardless of the treatment lines. However, the optimal time to initiate the PD-1/PD-L1 inhibitors requires further study. The longest DOR reached 21.65 m for atezolizumab as a second-line or subsequent treatment.

The addition of other therapies, such as chemotherapy, radiotherapy, or immunotherapy, is intended to improve the response efficiency of PD-1/PD-L1 monotherapy in clinical trials (105, 106). We found that being combined with chemotherapy (I+C) could significantly improve the ORR compared to monotherapy (I) by 29% (46.81% vs. 17.75%), while the addition of other immunotherapies (I+O) reduced the ORR compared with monotherapy (I) by 5% (12.25% vs. 17.75%). This difference might be partly due to the cytotoxicity of chemotherapy, which enabled immune cells to be activated by the increased antigens released by tumor cells. I+C (1.56 m) also appeared to reduce the time to response with a shorter TTR compared to I+O (1.97 m) and I (2.11 m), although there were no significant differences among the three groups. However, the addition of chemotherapy (I+C) significantly shortened the DOR compared to I by 4.3 m, but there was no pooled DOR of I+O due to the unavailability of relevant studies. Chemotherapy drugs can damage immune cells while killing tumor cells, which may be one reason for the shorter duration. Thus, the combination of immunotherapy and other therapies should be further studied in terms of treatment dosing, cycle, and medication order, so as to find the best way to achieve a synergistic effect.

We divided PD-1/PD-L1 inhibitors into PD-1 and PD-L1 groups to observe their response efficacy according to their different functional targets. PD-1 inhibitors were associated with better ORR (21.65% vs. 17.60%) and DOR (11.26 m vs. 10.03 m) compared to PD-L1 inhibitors. Among all the PD-1/PD-L1 inhibitors, atezolizumab had the best ORR (25.87%) and DOR (13.34 m), while durvalumab had the poorest ORR (13.20%) and DOR (7.40 m). These results demonstrate that the efficiency of different drugs could vary greatly even when they act on the same target, which should be considered carefully when selecting drugs in the clinic. As for TTR, there were no significant differences among different cancer types, treatment lines, drug combinations, or therapeutic regimens. The median TTR was about 2 months.

### Strengths and Limitations

A strength of this work is that we estimated the response efficacy of nearly 100 clinical trials and comprehensively analyzed their different clinical situations. We collected all available high-quality clinical trials for PD-1/PD-L1 from phases I to III. Thus, this meta-analysis can overcome the problem of inadequate power of each individual trial by pooling data together and minimizing inter-study heterogeneity. Furthermore, it is difficult to analyze TTR or DOR because a majority of clinical trials only provided the median and 95% CI without showing hazard ratios (HR) or individual patient data. Thus, we used a method of estimating the mean and standard deviation based on the median, range, and sample size. It has been reported that quantitative data without available means and standard deviations can be used in a meta-analysis without loss of accuracy (6, 7).

There are several limitations in this meta-analysis. First, the analysis was based on published results rather than on individual patient data. The estimation of means by using the median values will inevitably reduce precision. Second, the heterogeneity of many indicators can cause inaccuracy and publication biases existed among some outcomes. Third, we still lacked data from head-to-head comparisons, although the subgroups were divided in the meta-analysis. Therefore, caution should be exercised in interpreting the results of this study. Further prospective, randomized control trials are warranted for validation.

## Conclusions

This systematic meta-analysis determined the response efficacy of PD-1/PD-L1 inhibitors with different cancer types, treatment lines, drug combinations, and therapeutic regimens. PD-1/PD-L1 inhibitors are promising treatment modalities for various cancers with the potential for long-term clinical benefits. This large-scale meta-analysis of response efficacy can serve as a reference for clinicians to make evidence-based decisions.

## Data Availability Statement

The original contributions presented in the study are included in the article/**Supplementary Material**. Further inquiries can be directed to the corresponding author.

## Author Contributions

YH conceived and designed research. Data collection and extraction was performed by SC, ZZ, and XZ, and verified by PC. Statistical analysis was performed by HT, SZ, DH, ZH, ZW, and JM. ZL, JW, and YQ participated in drafting article. All authors contributed to the article and approved the submitted version.

## Conflict of Interest

The authors declare that the research was conducted in the absence of any commercial or financial relationships that could be construed as a potential conflict of interest.
