# Supplementary material for: Response Efficacy of PD-1 and PD-L1 Inhibitors in Clinical Trials: A Systematic Review and Meta-Analysis
Source: Front Oncol. 2021 Apr 16;11:562315. doi: 10.3389/fonc.2021.562315 (PMC8085334; doi:10.3389/fonc.2021.562315)
Supplement: Supplementary file 1 [file DataSheet_1.doc]

**Figure S1. Risk of bias summary of randomized controlled trials. + low risk, ? unclear risk, - high risk.**


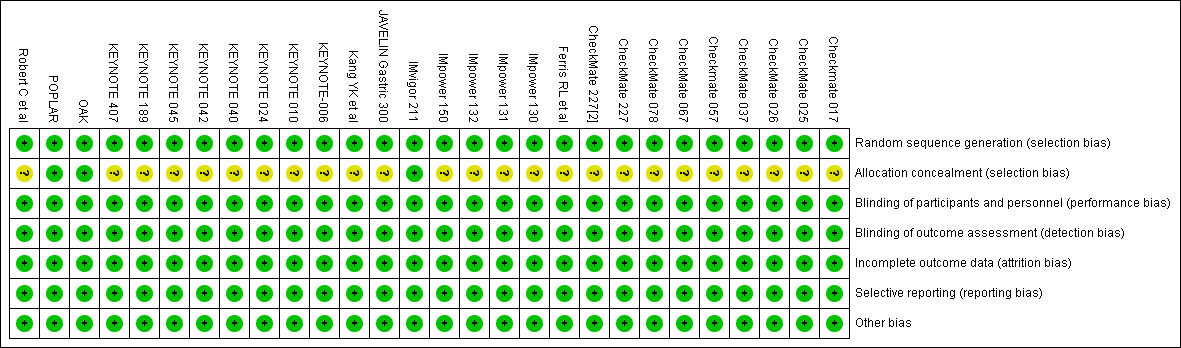


**Figure S2. Funnel plots of ORR by cancer type. (A), treatment line (B), drug combination (C), and therapeutic regimen (D).**

**Figure S3. Funnel plots of TTR by cancer type. (A), treatment line (B), drug combination (C), and therapeutic regimen (D).**

**Supplementary Figure S4. Funnel plots of DOR by cancer type. (A), treatment line (B), drug combination (C), and therapeutic regimen (D).**

| Outcomes | Included study numbers | Begg's tests (P) | Egger’s tests (P) |
| --- | --- | --- | --- |
| ORR by cancer type | 112 | 0.104 | 0.019 |
| ORR by treatment line | 84 | 0.184 | 0.065 |
| ORR by drug combination | 108 | 0.111 | 0.033 |
| ORR by therapeutic regimen | 109 | 0.112 | 0.032 |
| TTR by cancer type | 80 | 0.002 | 0.001 |
| TTR by treatment line | 58 | 0.573 | 0.102 |
| TTR by drug combination | 79 | 0.001 | 0.001 |
| TTR by therapeutic regimen | 80 | 0.001 | 0.002 |
| DOR by cancer type | 44 | 0.172 | 0.000 |
| DOR by treatment line | 35 | 0.733 | 0.002 |
| DOR by drug combination | 45 | 0.395 | 0.001 |
| DOR by therapeutic regimen | 45 | 0.270 | 0.001 |

**Table S2. Publication bias of different outcomes**
